# ORF3a-Mediated Incomplete Autophagy Facilitates Severe Acute Respiratory Syndrome Coronavirus-2 Replication

**DOI:** 10.3389/fcell.2021.716208

**Published:** 2021-07-27

**Authors:** Yafei Qu, Xin Wang, Yunkai Zhu, Weili Wang, Yuyan Wang, Gaowei Hu, Chengrong Liu, Jingjiao Li, Shanhui Ren, Maggie Z. X. Xiao, Zhenshan Liu, Chunxia Wang, Joyce Fu, Yucai Zhang, Ping Li, Rong Zhang, Qiming Liang

**Affiliations:** ^1^Research Center of Translational Medicine, Shanghai Institute of Immunology, Shanghai Children’s Hospital, Shanghai Jiao Tong University School of Medicine, Shanghai, China; ^2^Key Laboratory of Medical Molecular Virology (MOE/NHC/CAMS), School of Basic Medical Science, Shanghai Medical College, Biosafety Level 3 Laboratory, Fudan University, Shanghai, China; ^3^Faculty of Medicine, University of Alberta, Edmonton, AB, Canada; ^4^Department of Critical Care Medicine, Shanghai Children’s Hospital, Shanghai Jiao Tong University, Shanghai, China; ^5^Department of Statistics, University of California, Riverside, Riverside, CA, United States; ^6^Key Laboratory for Food Microbial Technology of Zhejiang Province, Zhejiang Gongshang University, Hangzhou, China; ^7^Key Laboratory of Cell Differentiation and Apoptosis of Chinese Ministry of Education, Shanghai Jiao Tong University School of Medicine, Shanghai, China; ^8^State Key Laboratory of Microbial Metabolism, Shanghai Jiao Tong University, Shanghai, China

**Keywords:** SARS-CoV-2, autophagy, ORF3a, UVRAG, COVID-19

## Abstract

Severe Acute Respiratory Syndrome Coronavirus-2 (SARS-CoV-2) is the causative agent for the coronavirus disease 2019 (COVID-19) pandemic and there is an urgent need to understand the cellular response to SARS-CoV-2 infection. Beclin 1 is an essential scaffold autophagy protein that forms two distinct subcomplexes with modulators Atg14 and UVRAG, responsible for autophagosome formation and maturation, respectively. In the present study, we found that SARS-CoV-2 infection triggers an incomplete autophagy response, elevated autophagosome formation but impaired autophagosome maturation, and declined autophagy by genetic knockout of essential autophagic genes reduces SARS-CoV-2 replication efficiency. By screening 26 viral proteins of SARS-CoV-2, we demonstrated that expression of ORF3a alone is sufficient to induce incomplete autophagy. Mechanistically, SARS-CoV-2 ORF3a interacts with autophagy regulator UVRAG to facilitate PI3KC3-C1 (Beclin-1-Vps34-Atg14) but selectively inhibit PI3KC3-C2 (Beclin-1-Vps34-UVRAG). Interestingly, although SARS-CoV ORF3a shares 72.7% amino acid identity with the SARS-CoV-2 ORF3a, the former had no effect on cellular autophagy response. Thus, our findings provide the mechanistic evidence of possible takeover of host autophagy machinery by ORF3a to facilitate SARS-CoV-2 replication and raise the possibility of targeting the autophagic pathway for the treatment of COVID-19.

## Introduction

Severe Acute Respiratory Syndrome Coronavirus-2 (SARS-CoV-2) is a novel coronavirus confirmed as the causative agent of coronavirus disease 2019 (COVID-19; Wu et al., 2020; Zhou et al., 2020). As of 27 May 2021, there have been more than 169 million confirmed cases of SARS-CoV-2 infection with more than 3.5 million deaths attributed to the virus in 235 countries and territories. The clinical presentation of symptoms of COVID-19 ranges from asymptomatic to acute respiratory distress syndrome. The majority of cases will be mild to moderate but individuals with underlying comorbidities are at higher risk for developing more serious complications including respiratory failure, shock, and multiorgan system dysfunction (Huang et al., 2020). While there continues to be unprecedented collaboration to facilitate the development of therapeutic neutralizing antibodies, vaccines, and small molecule drugs against COVID-19 (Florindo et al., 2020), only one FDA-approved drug [Veklury (Remdesivir)] is currently available and there is an urgent need to better understand how SARS-CoV-2 manipulates the host responses.

Severe Acute Respiratory Syndrome Coronavirus-2 is an enveloped, positive-sense, single-stranded RNA virus that engages human angiotensin-covering enzyme 2 (hACE2) to mediate host cell entry in the nasal passage, respiratory tract, and intestine (Hoffmann et al., 2020a). Infection with SARS-CoV-2 can lead to excessive production of pro-inflammatory cytokines and dysregulation of type I interferon response (Blanco-Melo et al., 2020; Hadjadj et al., 2020; Zhang et al., 2020). Quantitative proteomic analysis of lung epithelial (A549) cells infected with SARS-CoV-2 and peripheral blood mononuclear cell (PBMC) specimens of COVID-19 patients revealed perturbations of a broad range of cellular signaling pathways, including macroautophagy (Stukalov et al., 2021). Macroautophagy, hereafter referred to as autophagy, is a critical house-keeping process involving the formation of double-membrane autophagosomes that later fuse with lysosomes to degrade and recycle damaged organelles, unused proteins, and invading pathogens (Dikic and Elazar, 2018). In mammalian cells, this process is orchestrated by Beclin 1 (Levine et al., 2015), a scaffolding protein that regulates the lipid kinase Vps34 (PI3KC3) and interacts with several cofactors/adaptor proteins (Atg14 or UVRAG) to promote the formation of mutually exclusive Beclin 1-Vsp34 subcomplexes with distinct functions (Dikic and Elazar, 2018). The PI3KC3-C1 (Beclin 1-Vps34-Atg14) mainly positively regulates autophagy by promoting autophagosome formation while the PI3KC3-C2 (Beclin 1-Vps34-UVRAG) accelerates autophagosome maturation by promoting the fusion of autophagosomes with lysosomes to form autolysosomes (Levine et al., 2015). HOPS complexes and SNARE complexes are also involved in autophagosome maturation (Zhao and Zhang, 2019), and abrogation of either one of these three complexes will impair autophagosome maturation.

Autophagy is an important homeostatic mechanism for cell protection and is evolutionarily conserved from yeast to higher eukaryotes; however, a subset of viruses have evolved mechanisms to subvert and hijack the autophagic pathway to benefit their replication (Dong and Levine, 2013; Choi et al., 2018). For example, Zika virus utilizes NS4A and NS4B to inhibit the Akt-mTOR signaling pathway, leading to aberrant activation of autophagy and increased viral replication (Liang et al., 2016); Kaposi’s sarcoma-associated herpesvirus vBcl2, vFLIP, and K7 proteins serve as anti-autophagy molecules to inhibit autophagosome formation or maturation via targeting Beclin 1, Atg3, and Rubicon, respectively (Liang et al., 2008a, 2013; Lee et al., 2009; Gao et al., 2016); and herpes simplex virus 1 encodes protein ICP34.5 which binds to Beclin 1 and inhibits its autophagy function (Orvedahl et al., 2007). Studies on other beta-coronaviruses show that these viruses modulate autophagy (Miller et al., 2020; Zhao et al., 2021) but it is unknown whether viral evasion of autophagy is important in SARS-CoV-2 infection.

In this study, we found that SARS-CoV-2 infection or ORF3a expression triggers incomplete autophagy, resulting in elevated autophagosome formation but blockage of autophagosome maturation. By yeast two-hybrid screening, we found that SARS-CoV-2 ORF3a targets autophagy protein UVRAG to modulate Beclin 1 complexes by disrupting PI3KC3-C2 (Beclin 1-Vps34-UVRAG) but facilitating PI3KC3-C1 (Beclin 1-Vps34-Atg14). Importantly, SARS-CoV ORF3a does not share the autophagy modulatory activity with SARS-CoV-2 ORF3a. Our study not only highlights the role of ORF3a in autophagy modulation during SARS-CoV-2 infection but also raises the possibility of targeting the cellular autophagy for blocking SARS-CoV-2 replication.

## Results

### SARS-CoV-2 Infection Induces Incomplete Autophagy

**FIGURE 1:**
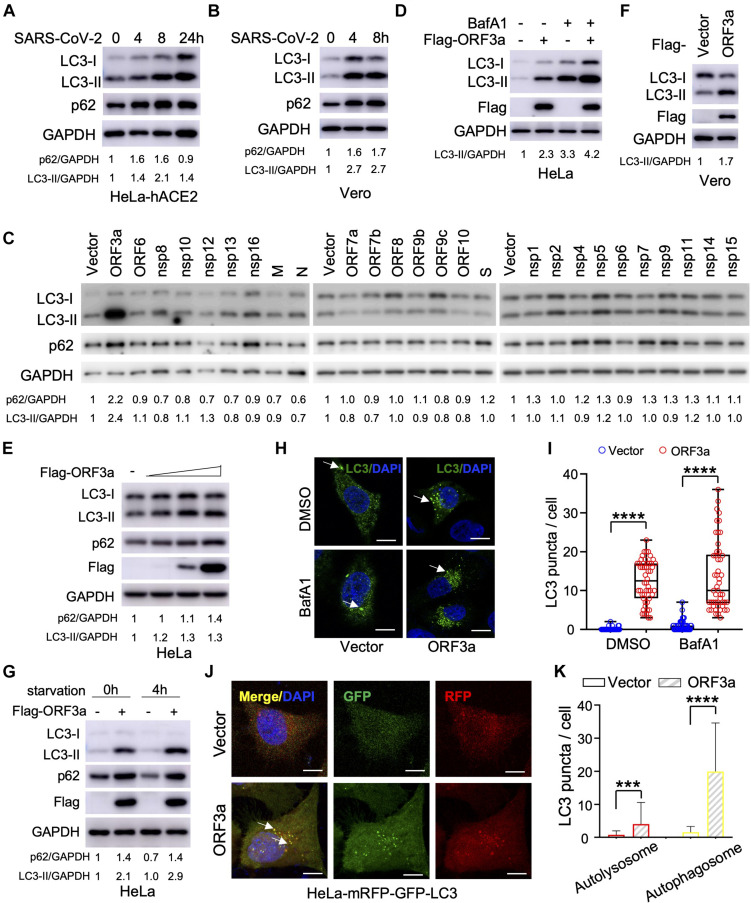
Severe Acute Respiratory Syndrome Coronavirus-2 (SARS-CoV-2) ORF3a triggers incomplete autophagy. **(A–B)** SARS-CoV-2 infection triggers incomplete autophagy. HeLa-hACE2 **(A)** or Vero-E6 **(B)** cells were infected with SARS-CoV-2 (MOI = 1) and cell lysates were collected at indicated time point post-infection for immunoblotting (IB) with indicated antibodies. **(C)** Screening of SARS-CoV-2-encoded proteins for LC3-I to LC3-II conversion in HeLa cells. **(D,E)** SARS-CoV-2 ORF3a induces incomplete autophagy. HeLa-vector and HeLa-ORF3a stable cells were treated with or without BafA1 (100 nM) for 4 h and the cell lysates were collected for IB with indicated antibodies **(D)**. HeLa cells were transfected with increasing amount ORF3a expressing plasmid and the cell lysates were collected for IB with indicated antibodies at 48 post-transfection **(E)**. **(F)** ORF3a expression modulates autophagy in Vero-E6 cells. **(G)** ORF3a blocks SQSTM1/p62 degradation upon starvation. HeLa-vector or HeLa-ORF3a stable cells were treated by serum starvation for 4 h and the cell lysates were collected for IB with indicated antibodies. **(H,I)** SARS-CoV-2 ORF3a induces LC3 puncta formation. HeLa-vector or HeLa-ORF3a cell line were treated with BafA1 (100 nM) and endogenous LC3 puncta were immunostained **(H)** and quantified **(I)**. Scale bar, 15 μm. Arrow: representative autophagosomes. **(J,K)** HeLa-mRFP-GFP-LC3 stable cells were transfected with ORF3a or vector control and transfected cells were fixed and the LC3 puncta were captured **(J,K)** quantified as indicated. Yellow puncta, autophagosomes; Red puncta, autolysosomes; Arrow, representative autophagosomes. p62/GAPDH or LC3-II/GAPDH levels were quantified by the band intensity in (A–G). Similar results were obtained by three independent experiments. Mean ± SEM; *n* = 50; ***p < 0.001 and ****p < 0.0001 by Student’s *t* test in panels **(I,K)**.

Severe Acute Respiratory Syndrome Coronavirus-2 can infect multiple hACE2-expressing cell lines and in refractory cell lines after exogenous hACE2 expression; however, it is unclear if SARS-CoV-2 infection modulates cellular autophagy response. To evaluate autophagic activity upon SARS-CoV-2 infection, we measured microtubule-associated protein light chain 3 (LC3) conversion (LC3-I to lipidated LC3-II), which is correlated with the formation of autophagosomes and is widely used as a marker to monitor autophagy (Klionsky et al., 2016). However, LC3-II is degraded together with the contents of the autophagosome and increased LC3-II may reflect either increased autophagosome formation or, alternatively, decreased autolysosome degradation (i.e., due to impairment of fusion between autophagosomes and lysosomes). Thus, it is important to measure SQSTM1/p62 turnover to evaluate autophagic flux (Klionsky et al., 2016). In stable hACE2 expressing HeLa cells infected with SARS-CoV-2 strain SH01, we detected dramatic conversion of LC3-I to LC3-II in the course of SARS-CoV-2 infection (Figure 1A), leading to accumulation of lipidated LC3-II. In addition, SQSTM1/p62 increased during the course of SARS-CoV-2 infection (Figure 1A), which strengthens the interpretation that infection blocked autophagosome-lysosome fusion and infected cells cannot form autolysosomes that degrade or recycle their contents. Similar LC3-I to LC3-II conversion and SQSTM1/p62 stability were also observed in infected Vero-E6 cells (Figure 1B), suggesting SARS-CoV-2 induces incomplete autophagy in infected cells.

### SARS-CoV-2 ORF3a Expression Induces Incomplete Autophagy

Since SARS-CoV-2 infection triggers an incomplete autophagy response in multiple cell lines, we attempted to determine which viral components could modulate the cellular pathways that control this process. The RNA genome of SARS-CoV-2 encodes 28 viral proteins, including 16 non-structural proteins (nsp1 to nsp16), four structural proteins [glycoprotein spike (S), membrane (M), envelope (E), and nucleocapsid (N)], and 8 accessory proteins (ORF3a, ORF6, ORF7a, ORF7b, ORF8, ORF9b, ORF9c, and ORF10). We screened each individual SARS-CoV-2 gene for LC3 conversion in HeLa cells and found that ORF3a expression resulted in a significant increase in the conversion of LC3-I to LC3-II (Figure 1C). However, other viral proteins had little or no effect (Figure 1C). Similar findings were recapitulated when ORF3a was expressed in HeLa cells in the presence and absence of bafilomycin A1 (Figures 1D,E), suggesting ORF3a itself can efficiently induce autophagosome accumulation. Similarly, ORF3a expression facilitated LC3-I to LC3-II conversion in Vero-E6 cells (Figure 1F). We also observed that SARS-CoV-2 ORF3a expression increased the SQSTM1/p62 level (Figure 1C), indicating the fusion step between autophagosome and lysosome (autophagosome maturation) is blocked. Indeed, starvation-mediated SQSTM1/p62 degradation was also abolished by ORF3a in HeLa cells (Figure 1G), indicating that ORF3a expression activates autophagy but suppressing autophagic degradation. Consistently, expression of ORF3a led to dramatically elevation of LC3 puncta per cell in HeLa cells with or without Bafilomycin A1 treatment (Figures 1H,I), confirming the efficient induction of autophagosome formation. In addition, we utilized tandem fluorescent-tagged LC3 (mRFP-GFP-LC3) to differentiate between autophagosomes and autolysosomes upon SARS-CoV-2 ORF3a expression. Since GFP is unstable in the acidic environment of lysosome, unmatured autophagosomes, and matured autolysosomes are labeled by yellow LC3 puncta (GFP^+^mRFP^+^) or red LC3 puncta (GFP^–^mRFP^+^), respectively (Kimura et al., 2007). Although SARS-CoV-2 ORF3a increased matured autolysosome (red puncta), it promoted unmatured autophagosome (yellow puncta) to a higher level (Figures 1J,K), indicating that ORF3a triggers autophagy but also inhibits the fusion between autophagosomes and lysosomes. Taken together, our findings suggest that ORF3a efficiently triggers incomplete autophagy in multiple cell lines, which phenocopies SARS-CoV-2 infection.

### SARS-CoV-2 ORF3a Interacts With UVRAG to Reshape PI3KC3 Complexes

**FIGURE 2:**
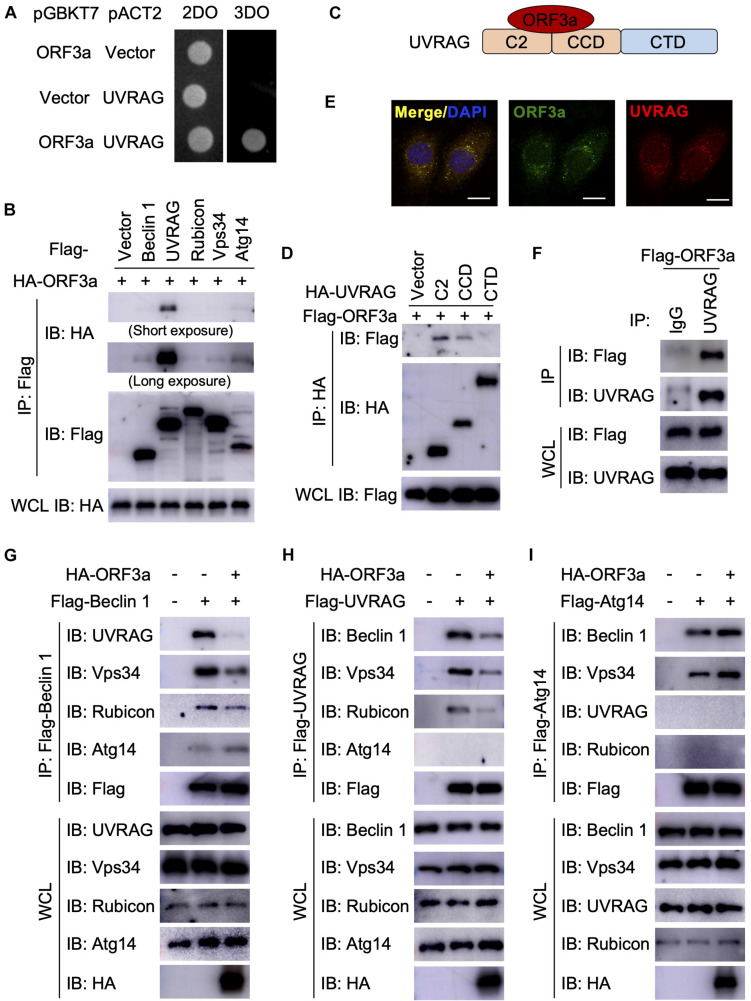
Severe Acute Respiratory Syndrome Coronavirus-2 ORF3a targets UVRAG to modulate PI3KC3 complex formation. **(A)** SARS-CoV-2 ORF3a interacts with UVRAG in yeast two-hybrid system. **(B)** SARS-CoV-2 ORF3a binds to UVRAG in HEK293T cells. HEK293T cells were co-transfected with indicated plasmids and cell lysates were collected and subjected to immunoprecipitation (IP) and IB with indicated antibodies at 48 h post-transfection. **(C)** Schematic diagram of SARS-CoV-2 ORF3a-UVRAG interaction. **(D)** The C2 and CCD of UVRAG bind to SARS-CoV-2 ORF3a. **(E)** SARS-CoV-2 ORF3a co-localizes with endogenous UVRAG. HeLa-ORF3a stable cells were subjected to immunostaining with antibodies against Flag or UVRAG. Scale bar, 15 μm. **(F)** SARS-CoV-2 ORF3a binds to endogenous UVRAG in HEK293T cells. **(G–I)** SARS-CoV-2 ORF3a blocks PI3KC3-C2 but facilitates PI3KC3-C1. HEK293T cells were co-transfected with indicated plasmids and cell lysates were collected and subjected to IP and IB with indicated antibodies at 48h post-transfection.

Next, we sought to determine the mechanism through which SARS-CoV-2 ORF3a induces incomplete autophagy. In recent months, four independent virus-host interactomes analyzed by network based-approach provided insights into viral pathogenesis (Gordon et al., 2020a,b; Li et al., 2021; Stukalov et al., 2021), but it remains unclear how SARS-CoV-2 interacts with the cellular autophagy machinery to trigger incomplete autophagy. Using yeast two-hybrid screening with SARS-CoV-2 ORF3a as a bait, we captured a novel protein-protein interaction with UVRAG, a key autophagy regulator that activates PI3KC3 complex to promote autophagosome maturation and suppresses the proliferation of human colon cancer cells (Liang et al., 2006, 2008b). SARS-CoV-2 ORF3a interacted with UVRAG in yeast (Figure 2A). Among Beclin 1 complex components, UVRAG showed the strongest binding affinity to ORF3a in HEK293T cells (Figure 2B). Detailed mapping suggested that ORF3a interacted with the N-terminal C2 and coiled coil domain (CCD) of UVRAG (Figures 2C,D). The CCD provides a platform for many important protein interactions and is required for UVRAG binding to Beclin 1 (Liang et al., 2006). ORF3a also bound to and co-localized with endogenous UVRAG in HeLa cells (Figures 2E,F), further confirming UVRAG is the potential host target for SARS-CoV-2 ORF3a. Beclin 1 is an essential autophagy protein that orchestrates the assembly of functionally distinct PI3KC3 multiprotein complexes that regulate autophagosome formation and maturation (Levine et al., 2015). The core complex consists of Beclin 1, Vps34, and Vps15 which interacts with key modulators Atg14 and UVRAG to form mutually exclusive Atg14- or UVRAG-containing PI3KC3 subcomplexes that positively regulates autophagy by promoting early-stage autophagosome formation and late-stage autophagosome maturation, respectively (Levine et al., 2015). Since ORF3a binds to UVRAG and triggers incomplete autophagy, we speculated that ORF3a expression may lead to aberrant redistribution of Atg14- or UVRAG-containing PI3KC3 complexes and disrupt the balance between autophagosome formation and maturation. To test our hypothesis, we purified the Beclin 1 complex, UVRAG complex, and Atg14 complex, respectively, in the presence and absence of SARS-CoV-2 ORF3a. In line with previous reports, Atg14 and UVRAG were not present in the same complex and Rubicon is only associated with PI3KC3-C2 in the absence of ORF3a (Matsunaga et al., 2009; Zhong et al., 2009; Figures 2G–I). In contrast, the presence of ORF3a significantly reduced the interaction between UVRAG and Beclin 1 or other Beclin 1 complex components, such as Vps34 and Rubicon, suggesting that PI3KC3-C2 formation is disrupted by SARS-CoV-2 ORF3a (Figures 2G,H). Furthermore, SARS-CoV-2 ORF3a expression enhanced the interaction between Atg14 and Beclin 1, suggesting dissociation between Beclin 1 and UVRAG facilitated PI3KC3-C1 assembly (Figures 2G,I). Taken together, these findings demonstrate that the ORF3a-UVRAG interaction renders UVRAG less competitive than Atg14 for Beclin 1 binding and shifts the balance between PI3KC3-C1 and PI3KC3-C2, resulting in more efficient autophagosome formation but inhibition of autophagosome maturation.

### Autophagy Deficiency Decreases SARS-CoV-2 Replication

**FIGURE 3:**
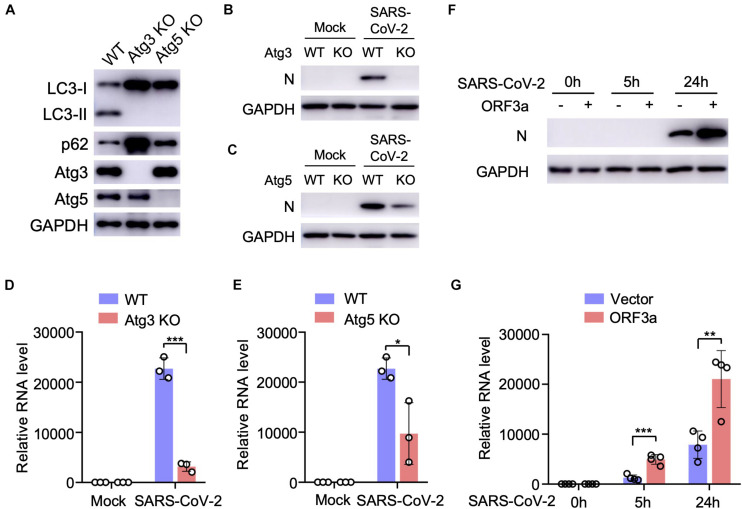
Cellular autophagy is required for efficient SARS-CoV-2 replication. **(A)** Genetic abrogation of Atg3 or Atg5 blocks autophagy response in MEF-hACE2 cell lines. **(B–E)** Atg3 or Atg5 KO reduces SARS-CoV-2 replication efficiency in MEF-hACE2 stable cells. MEF-hACE2^*WT*^, MEF-hACE2^*Atg*3 KO^, or MEF-hACE2^*Atg*5 KO^ were infected with SARS-CoV-2 (MOI = 1) and virus replication was examined by IB of viral N protein **(B,C)** or quantitative RT-PCR of viral transcripts **(D,E)**. **(F,G)** ORF3a expression facilitates SARS-CoV-2 replication. Calu3-vector or Calu3-ORF3a stable cells were infected with SARS-CoV-2 (MOI = 1) and virus replication was examined by IB of viral N protein **(F)** or quantitative RT-PCR of viral transcripts **(G)**. Mean ± SEM; *n* = 3 **(D,E)** or *n* = 4 **(G)**; ns, not significant, *p < 0.05; **p < 0.01; ***p < 0.001 by Student’s *t* test.

Since SARS-CoV-2 infection or ORF3a expression modulates cellular autophagy pathway, we next analyzed the effect of autophagy on SARS-CoV-2 replication using cell lines with genetic abrogation of autophagic essential genes. Atg3 and Atg5 are two indispensable proteins that mediate vesicle elongation during the autophagosome formation process and genetic knockout of Atg3 or Atg5 blocks LC3 conversion and SQSTM1/p62 degradation in MEFs (Dikic and Elazar, 2018; Figure 3A). Using MEF stable expressing hACE2 receptor, we investigated how abrogation of cellular autophagy response by Atg3 or Atg5 knockout affects SARS-CoV-2 replication. As shown in Figures 3B–E, the autophagy machinery is required for efficient SARS-CoV-2 replication, and infection of autophagy-deficient cells resulted a significant reduction in viral yield compared to yield in control cells, as shown by immunoblot analyses of viral N protein expression (Figures 3B,C) and quantitative RT-PCR of viral transcripts (Figures 3D,E). To determine whether ORF3a affects SARS-CoV-2 replication, we infected Calu3-vector and Calu3-ORF3a stable cells with SARS-CoV-2 and evaluated virus replication. Compared to vector control, ORF3a expression significantly increased SARS-CoV-2 replication (Figures 3F,G). Collectively, these results demonstrated that cellular autophagy response is required for efficient SARS-CoV-2 replication.

### SARS-CoV ORF3a Does Not Interact With UVRAG and Has No Effect on Autophagy

**FIGURE 4:**
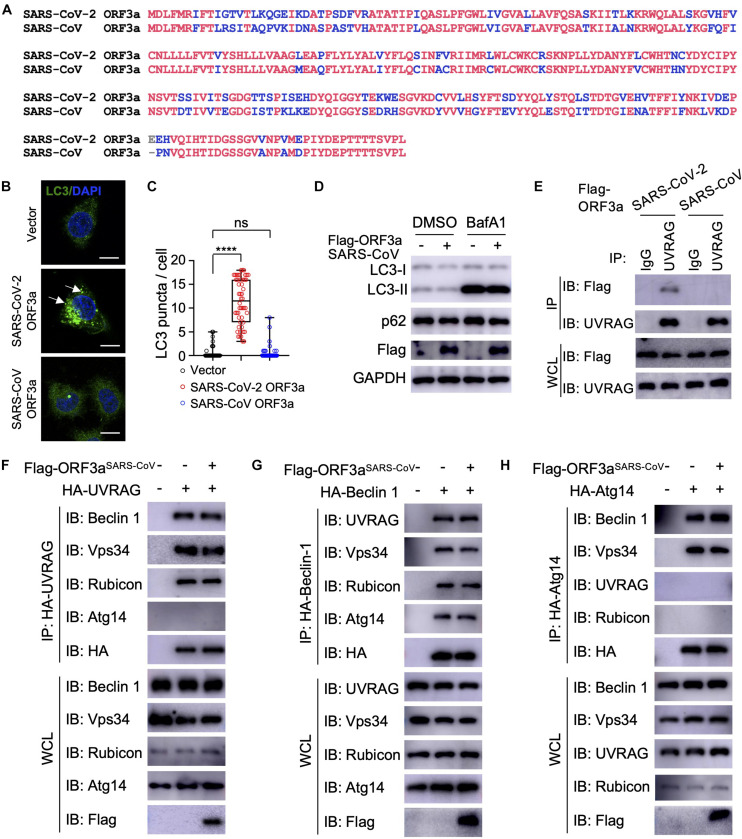
SARS-CoV ORF3a cannot induce autophagy. **(A)** Sequence alignment between SARS-CoV-2 ORF3a and SARS-CoV ORF3a. **(B–D)** SARS-CoV ORF3a does not affect autophagy. HeLa-vector, HeLa-ORF3a^*SARS*–*CoV*–2^, or HeLa-ORF3a^*SARS*–*CoV*^ cell lines were treated with BafA1 (100 nM) and endogenous LC3 puncta were immunostained **(B)** and quantified **(C)**. Scale bar, 15 μm. Arrow: representative autophagosomes. Mean ± SEM; *n* = 50; *ns* and ****p < 0.0001 by one-way ANOVA and Bonferroni’s *post hoc* test. HeLa-vector or HeLa-ORF3a^*SARS–CoV*^ cell lines were treated with BafA1 (100 nM) for 4 h and the cell lysates were collected for IB with indicated antibodies **(D)**. **(E)** ORF3a^*SARS–CoV–*2^ but not ORF3a^*SARS*–*CoV*^ interacts with endogenous UVRAG. HEK293T cells were transfected with Flag-ORF3a^*SARS*–*CoV*^ or Flag-ORF3a^*SARS–CoV–*2^ and cell lysates were collected and subjected to IP and IB with indicated antibodies at 48h post-transfection. **(F–H)** SARS-CoV ORF3a does not affect the formation of UVRAG complex **(F)**, Beclin 1 complex **(G)**, or Atg14 complex **(H)**. HEK293T cells were co-transfected with indicated plasmids and cell lysates were collected and subjected to IP and IB with indicated antibodies at 48 h post-transfection.

The genome of SARS-CoV-2 is closely related to SARS-CoV, the first deadly coronavirus that caused the SARS epidemic in 2002–2003 (Zhong et al., 2003). Since ORF3a^*SARS*–*CoV*^ shares 72.7% amino acid sequence identity to ORF3a^*SARS–CoV–*2^ (Figure 4A), we sought to determine whether the ORF3a homolog from SARS-CoV could trigger similar incomplete autophagy. We generated a stable HeLa cell line that expresses ORF3a^*SARS*–*CoV*^ and examined its effects on autophagy markers, including LC3-I to LC3-II conversion and LC3 puncta formation. Unlike ORF3a^*SARS–CoV–*2^, ORF3a^*SARS*–*CoV*^ expression led to neither dramatic increase in the amount of LC3-II nor accumulation of LC3 puncta (Figures 4B–E), indicating ORF3a^*SARS*–*CoV*^ cannot efficiently induce the formation of autophagosomes or suppress autophagosome maturation. Furthermore, ORF3a^*SARS*–*CoV*^ did not interact with endogenous UVRAG (Figure 4F) and could not modulate the formation of PI3KC3-C1 or PI3KC3-C2 (Figures 4F–H). These results show that ORF3a-mediated modulation of autophagy response is a unique signature of SARS-CoV-2 infection and may play an important role in controlling viral replication and pathogenesis.

## Discussion

**FIGURE 5:**
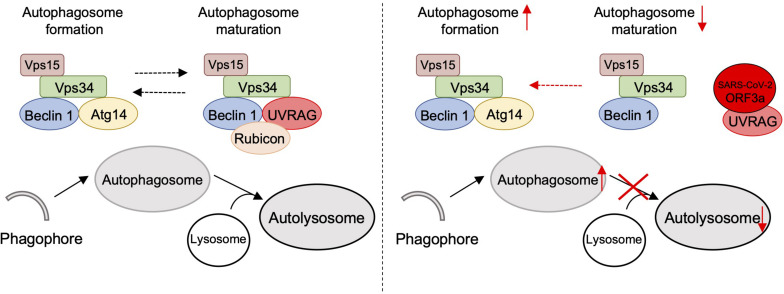
Schematic diagram of SARS-CoV-2 ORF3a-mediated incomplete autophagy. SARS-CoV-2 ORF3a interacts with UVRAG to positively regulate PI3KC3-C1 but suppress the PI3KC3-C2, leading to elevated autophagosome formation but blockage of autophagosome maturation.

Many coronaviruses trigger autophagy in infected cells (Brest et al., 2020; Mijaljica and Klionsky, 2020), however, how SARS-CoV-2 modulates cellular autophagy and whether autophagy affects SARS-CoV-2 replication remain elusive. In this study, we found that ORF3a triggers incomplete cellular autophagy to generate double-membrane autophagosome vesicles, which are required for efficient SARS-CoV-2 replication. Mechanistically, SARS-CoV-2 ORF3a interacts with UVRAG to positively regulate PI3KC3-C1 but suppress the PI3KC3-C2, thus elevating autophagosome formation and blocking autophagosome maturation (Figure 5). Knockout of Beclin 1 or Vps15 fully blocked SARS-CoV-2 replication in Huh7.5.1 cells (Wang et al., 2021), supporting the role of autophagosome formation is required for SARS-CoV-2 replication. Since ORF3a silenced UVRAG activity on autophagy by inhibiting PI3KC3-C2, knockout of UVRAG had little effect on SARS-CoV-2 replication (Wang et al., 2021). Recent study showed that SARS-CoV-2 ORF3a blocks autophagosome maturation by blocking HOPS-mediated SNARE complex assembly (Miao et al., 2021; Zhang et al., 2021), suggesting ORF3a may target multiple protein complexes required for autophagosome-lysosome fusion to facilitate SARS-CoV-2 replication. Given autophagy’s role in the innate antiviral immune response to restrict infecting pathogens, it is not surprising that viruses are in a constant arms race to remodel the autophagic membranes for their own benefit during replication (Choi et al., 2018). SARS-CoV-2 ORF3a hijacks the autophagy machinery to generate double-membrane autophagosome vesicles to facilitate viral replication but arrests the autophagosomes prior to lysosome fusion to avoid succumbing to lysosomal degradation. Interestingly, although ORF3a^*SARS*–*CoV*^ shares 72.7% amino acid identity with the ORF3a^*SARS–CoV–*2^, the former had no effect on the formation of double-membrane autophagosome vesicles. Coronaviruses are known to rely on the formation of convoluted autophagosome-like double membrane vesicles for optimal replication (Brest et al., 2020; Wolff et al., 2020), and ORF3a-mediated autophagosome formation may be one source of these double-membrane replication organelles. Although SARS-CoV ORF3a has no impact on cellular autophagy, nsp6 of SARS-CoV has been shown to limit autophagosome expansion (Cottam et al., 2014). Actually, nsp6 homologs from multiple coronaviruses, such as avian coronavirus, mouse hepatitis virus, SARS-CoV, and SARS-CoV-2, affect autophagosome membrane expansion, resulting in small autophagosome (Cottam et al., 2014). Besides nsp6 and ORF3a, ORF7a, and M of SARS-CoV-2 also inhibit autophagy at different levels, while within SARS-CoV-2-encoded proteins, ORF3a shows the most potent capability on autophagy modulation (Miao et al., 2021; Zhang et al., 2021). Manipulation of cellular autophagy pathway is a common signature of coronaviruses and ORF3a is a potential target to limit SARS-CoV-2 replication.

Veklury (Remdesivir) has just been approved for the treatment of SARS-CoV-2 infection and several preclinical investigations repurposing several FDA-approved drugs are underway. Since elevated autophagy facilitates coronaviruses replication efficiency (Brest et al., 2020), the drugs, such as rapamycin (Stukalov et al., 2021), that enhance autophagy should be avoided throughout the course of treatment. Antimalarial drugs chloroquine (CQ) and hydroxychloroquine are lysosomotropic agents that inhibit the pH-dependent replication steps in several viruses and many publications have urged the use of CQ as a potential treatment for COVID-19 based on *in vitro* considerations (Savarino et al., 2003; Colson et al., 2020; Devaux et al., 2020; Gao et al., 2020; Maisonnasse et al., 2020). However, there are still discrepancies and concerns on the sensitivity and therapeutic range of CQ as its effectiveness on limiting SARS-CoV-2 replication did not extend to TMPRSS2-expressing human lung cells (Hoffmann et al., 2020b), suggesting the cell specific responses may exist. In addition, CQ-mediated inhibition of cellular autophagy response may also contribute to its effects on SARS-CoV-2 (Klionsky et al., 2016). In summary, our work highlights the mechanism of how SARS-CoV-2 co-opts the autophagy pathway to enhance its own replication and spread, and raises the possibility of targeting the autophagic pathway for the treatment of COVID-19.

## Materials and Methods

### Viruses, Plasmids, and Cell Culture

Severe Acute Respiratory Syndrome Coronavirus-2 strain SH01 (GenBank: MT121215.1) was described previously (Zhu et al., 2021). SARS-CoV-2 stocks were propagated in Vero-E6 cells and the titer of SARS-CoV-2 stocks were determined by standard plaque assay on Vero-E6 cells as described previously (Liang et al., 2014). Experiments related to SARS-CoV-2 infection are performed in BSL-3 laboratory in Fudan University.

Severe Acute Respiratory Syndrome Coronavirus-2 genes in pLVX-3xFlag-MCS-P2A-tagRFP (puro) have been described previously (Li et al., 2021). ORF3a^*SARS–CoV–2*^ were subcloned into pEF-MCS-3xHA, pCDH (Hygro), or pGBKT7 vectors. The ORF3a^*SARS–CoV*^ template was kindly provided by Dr. Peihui Wang (Shandong University, China) and cloned into pLVX vector. The constructs encoding Beclin-1, Atg14, Vsp34, UVRAG, and Rubicon have been previously described (Liang et al., 2013). All constructs were sequenced using an ABI PRISM 377 automatic DNA sequencer to verify 100% correspondence with the original sequence.

HEK293T (ATCC, #CRL-11268), HeLa (ATCC, #CCL-2), A549 (ATCC, #CCL-185), and MEF (wild-type, Atg3 KO, or Atg5 KO, gift from Dr. Qing Zhong at Shanghai Jiao Tong University School of Medicine, China) cells were maintained in Dulbecco’s modified Eagle’s medium (DMEM; Gibco-BRL) containing 4 mM glutamine and 10% FBS. Vero-E6 cells were cultured in DMEM with 5% FBS and 4 mM glutamine. Calu-3 cells were cultured in DMEM with 20% FBS, 2 mM glutamine, 10 mM HEPES, and MEM Non-Essential Amino Acids. Transient transfections were performed with Lipofectamine 3000 (Thermo Fisher Scientific, #3000015). HeLa stable cell lines expressing SARS-CoV-2 proteins were generated using a standard lentivirus infection and selection protocol with puromycin (2 μg/ml) or hygromycin (200 μg/ml) for the expression of SARS-CoV-2 genes (Liang et al., 2016). Due to the low expression levels of nsp3 or E in HeLa, we excluded these two viral proteins in autophagy phenotype screening.

### Detection of Autophagy

For the LC3 conversion assay, SARS-CoV-2-infected or ORF3a expressing cells were washed with cold PBS, lysed with 1% Triton X-100, and then subjected to immunoblot analysis (15% SDS-PAGE) with antibodies against LC3 (Cell Signaling, #3868) or SQSTM1/p62 (Cell Signaling, #39749). LC3-I is about 16 kD and lipidated LC3 (LC3-II) is about 14 kD. p62/GAPDH or LC3-II/GAPDH levels were quantified by the band intensity from immunoblotting. SARS-CoV-2-infected or ORF3a expressing cells were fixed and endogenous LC3 was detected by confocal microscope. Briefly, cells were fixed with 4% paraformaldehyde for 20 min, permeabilized with Triton X-100 (0.2%) for 10 min, blocked with 1% BSA for 30 min, and immunostained with a primary antibody against LC3 (Cell Signaling, #3868) overnight at 4°C, followed by a fluorescent secondary antibody for 2 h at room temperature. Nuclei were stained by DAPI (Thermo Fisher Scientific, #62248) and images of cells were collected with Leica TCS SP8 confocal microscope. To quantitate LC3-positive autophagosomes per cell, cells from five random fields (>50 cells) were counted. The number of LC3 puncta per cells was counted manually in three independent experiments. Similar results were obtained by three independent experiments.

### Immunoprecipitation and Immunoblot Analysis

For co-immunoprecipitation, 2 × 10^6^ HEK293T cells were transfected with 20 μg of plasmid at a confluency of 90% with Lipofectamine 3000 (Thermo Fisher Scientific, #3000015). The cells were washed twice with cold phosphate-buffered saline (PBS) and lysed in a whole cell lysis buffer (WCL) containing [50 mM *Tris*⋅HCl (pH 7.4), 150 mM NaCl, 1% NP-40, 1 mM EDTA, 10% glycerol, protease inhibitor cocktail (Roche)] for 20 min on ice at 48 h post-transfection. The cell lysates were then centrifuged at 15,000 rpm for 15 min and the clear supernatants were subjected to immunoprecipitation with anti-Flag M2 agarose resin (Sigma, #F2426) or anti-HA agarose resin (Sigma, #E6779) following the manufacturer’s instruction. After 4h incubation at 4°C, the beads were washed three times with WCL and twice with PBS, and then boiled with the 2 × loading buffer for 10 min. The immunoprecipitants were applied to standard immunoblotting analyses with indicated specific antibodies.

For immunoblotting, cell lysates were collected in WCL and separated by SDS-PAGE and transferred to PVDF membrane (Bio-Rad) by semi-dry transfer at 25 V for 30 min. All membranes were blocked in 5% milk in PBST and probed overnight with indicated antibodies in 5% BSA at 4°C. Primary antibodies included: mouse Flag (Sigma, #F1804), rat Flag-HRP (Biolegend, #637311), mouse HA (BioLegend, #901515), rabbit SQSTM1/p62 (Cell Signaling, #39749), rabbit LC3 (Cell Signaling, #3868), rabbit Beclin-1 (Cell Signaling, #3495), rabbit UVRAG (Cell Signaling, #13115), rabbit Atg14 (Cell Signaling, #96752), rabbit Rubicon (Cell Signaling, #8465), rabbit Vps34 (Cell Signaling, #4263), rabbit Atg3 (Cell Signaling, #3415), rabbit Atg5 (Cell Signaling, #12994), mouse SARS-CoV-2 N (GeneTex, #GTX635689), and mouse GAPDH (Santa Cruz, #365062). Appropriate HRP-conjugated secondary antibodies were incubated on membranes in 5% milk and bands were developed with ECL reagent (Thermo Scientific) and imaged on a Fuji LAS-4000 imager.

### Yeast Two-Hybrid Assay

Yeast two-hybrid interaction assay was performed as previously described (Liang et al., 2018; Li et al., 2021). Briefly, Y2HGold Yeast Strain (Clontech, #630498) that contains four integrated reporter genes under the control of three distinct Gal4-responsive promoters are used to detect two-hybrid interactions. pGBKT7 (DNA binding domain) and pACT2 (activation domain) constructs were simultaneously transformed using Yeastmaker^TM^ Yeast Transformation System 2 (Clontech, #63039) following the manufactory manual. Transformed yeast cells were washed by sterile water one time and resuspended in sterile water and spotted onto -2DO plates (SD/-Leu/-Trp dropout medium) to assess transformation efficiency and onto -3DO plates (SD/-Ade/-Leu/-Trp selection medium) to evaluate the potential interactions. Plates were incubated for 4–6 days at 30°C for the positive clone growth. All constructs were tested for autoactivating properties to confirm no autonomous activation on the reporter genes. All the positive interactions were confirmed by at least three technical replicates.

### RNA Extraction and Quantitative RT-PCR

Total RNA was isolated from cells with the RNeasy Mini Kit (Qiagen, #74106) and treated with RNase-free DNase according to the manufacturer’s protocol. All gene transcripts were quantified by quantitative PCR using qScript^TM^ One-Step qRT-PCR Kit (Quanta Biosciences, #95057-050) on CFX96 real-time PCR system (Bio-Rad). Primer sequences for qPCR were as follow: SARS-CoV-2-N forward: GACCCCAAAATCAGC GAAAT, SARS-CoV-2-N reverse: TCTGGTTACTGCCAGTTG AATCTG; 18S forward: GTAACCCGTTGAACCCCATT, 18S reverse: CCATCCAATCGGTAGTAGCG.

### Quantification and Statistical Analysis

All data were expressed as Mean ± SEM as indicated. Statistical significance across two groups was tested by Student’s *t*-test; one-way analysis of variance (ANOVA) followed by Bonferroni’s *post hoc* test were used to determine statistically significant differences between multiple groups. *P*-values of less than 0.05 were considered significant.

## Data Availability Statement

The original contributions presented in the study are included in the article/supplementary material, further inquiries can be directed to the corresponding authors.

## Author Contributions

QL, RZ, PL, and YCZ conceived of the research, designed the study, and wrote the manuscript. YQ, XW, CL, JL, ZL, and WW performed the experiments and analyzed the data. YKZ, YW, GH, and SR performed the SARS-CoV-2 infection experiments in BSL-3 Laboratory. MX helped with visual representation of data and edited the manuscript. All authors commented on the manuscript.

## Conflict of Interest

The authors declare that the research was conducted in the absence of any commercial or financial relationships that could be construed as a potential conflict of interest.

## Publisher’s Note

All claims expressed in this article are solely those of the authors and do not necessarily represent those of their affiliated organizations, or those of the publisher, the editors and the reviewers. Any product that may be evaluated in this article, or claim that may be made by its manufacturer, is not guaranteed or endorsed by the publisher.
